# Amyloid persistence in decellularized liver: biochemical and histopathological characterization

**DOI:** 10.3109/13506129.2015.1110518

**Published:** 2015-12-08

**Authors:** Giuseppe Mazza, J. Paul Simons, Raya Al-Shawi, Stephan Ellmerich, Luca Urbani, Sofia Giorgetti, Graham W. Taylor, Janet A. Gilbertson, Andrew R. Hall, Walid Al-Akkad, Dipok Dhar, Philip N. Hawkins, Paolo De Coppi, Massimo Pinzani, Vittorio Bellotti, P. Patrizia Mangione

**Affiliations:** ^a^ Institute for Liver and Digestive Health; ^b^ Wolfson Drug Discovery Unit, Centre for Amyloidosis and Acute Phase Proteins; ^c^ Centre for Biomedical Science, Division of Medicine, University College London, London, UK; ^d^ Stem Cells and Regenerative Medicine Section, Developmental Biology and Cancer Programme, UCL Institute for Child Health, Great Ormond Street Hospital, University College London, LondonUK; ^e^ Department of Molecular Medicine, Institute of Biochemistry, University of Pavia, Pavia, Italy; ^f^ Organ Transplantation Centre and Comparative Medicine Department, King Faisal Specialist Hospital, Riyadh, Saudi Arabia

**Keywords:** AA amyloidosis, AEF, amyloid, decellularized liver, scaffold

## Abstract

Systemic amyloidoses are a group of debilitating and often fatal diseases in which fibrillar protein aggregates are deposited in the extracellular spaces of a range of tissues. The molecular basis of amyloid formation and tissue localization is still unclear. Although it is likely that the extracellular matrix (ECM) plays an important role in amyloid deposition, this interaction is largely unexplored, mostly because current analytical approaches may alter the delicate and complicated three-dimensional architecture of both ECM and amyloid. We describe here a decellularization procedure for the amyloidotic mouse liver which allows high-resolution visualization of the interactions between amyloid and the constitutive fibers of the extracellular matrix. The primary structure of the fibrillar proteins remains intact and the amyloid fibrils retain their amyloid enhancing factor activity.

## Introduction

Amyloidosis is a group of protein misfolding disorders in which normal circulating proteins misfold and self-aggregate into fibrillar polymers that precipitate in the extracellular space [1]. The disease can be systemic when several organs and connective tissue matrix are widely involved, or localized if only a single anatomical site or organ system is affected. In many cases of systemic amyloidosis, amyloid deposition is most prominent in specific organs, such as the liver, the heart, the spleen or the nerves, and this dictates the specific clinical features. Although there is clinical and experimental evidence suggesting that some specific constituents of the extracellular matrix (ECM), such as proteoglycans may play a crucial role in determining the specificity of tissue localization of amyloid deposits [2], accurate analysis of the interaction between amyloid and the biochemical and biophysical structure of the ECM has been so far been limited by technical factors.

We speculated that the intimate relationship between ECM and amyloid could be more precisely characterized in amyloidotic tissue after cell removal. Decellularization of a biopsy of amyloidotic tissue was first carried out in a pioneering study by Bonsib and Plattner [3] in the attempt to improve the microscopic visualization of amyloid, however the decellularization of an entire amyloidotic organ has not previously been undertaken. Notably, successful creation of scaffolds for tissue regeneration has lately been achieved by decellularization of whole organs [4–7]. The architecture of the decellularized organ is maintained with the preservation of the fine 3D structure of the ECM and of the vascular, lymphatic and nervous networks [8]. Cells can be removed by mild procedures which preserve not only the 3D architecture but also the biochemical composition, the bioactivity and the physical properties of the ECM [9]. We have previously shown that a specific detergent-enzymatic treatment [10] preserves scaffold microarchitecture and microvascular networks allowing successful clinical transplantation of human tracheas [11].

Here, we report in mice with transgenic overexpression of mouse amyloid A (AA) [12] that hepatic AA amyloid deposits [12] persisted in the ECM after complete decellularization of the liver. The primary structure of the amyloid fibril protein was unaffected by the procedure and amyloid extracted from decellularized liver retained the amyloid enhancing factor (AEF) property that accelerates, *in vivo*, AA amyloid formation. In addition, scanning electron microscope (SEM) images of the scaffold of the decellularized amyloidotic organ provided a unique 3D visualization of the intimate relationship between the amyloid fibrils and the ECM network.

## Methods

### Transgenic mice

Amyloidosis was induced in transgenic mice, which express the amyloidogenic SAA1 isotype [13] (originally called SAA2 [14]) when treated with doxycycline, as previously described [12]. Briefly, AEF was injected into animals receiving 2 mg/ml doxycycline in their drinking water for 10 days after AEF administration. This protocol consistently results in extremely large amyloid loads [12].

### Harvest of organs for decellularization

Mice were killed by CO_2_ inhalation. The abdomen was cleaned with 70% ethanol and a U-shaped incision was performed to expose the abdominopelvic cavity. The abdominal inferior vena cava (IVC) and portal vein (PV) were identified and the PV was cannulated with a 24G cannula (Becton Dickinson, Franklin Lakes, NJ), which was secured in place with a 3-0 silk suture (Ethicon, Somerville, NJ). The abdominal IVC was sectioned and 200 U of heparin injected into the portal vein. The diaphragm was used as a holding point to release the whole liver from the supporting tissue. The whole procedure was carried out with special caution not to damage Glisson’s capsule, which surrounds the organ.

### Decellularization

Mouse livers were connected to a peristaltic pump via the cannulated PV and perfused with deionized water for 36 h at 4 °C. Livers were then transferred to room temperature and were perfused with 4% (w/v) sodium deoxycholate (SDC) (Sigma, St Louis, MO) for 6 h followed by perfusion of 500 kU/ml of DNase-I from bovine pancreas (Sigma, St Louis, MO) in 1 M sodium chloride (NaCl) for 3 h. The flow rate was kept at 4.5 ml/min for the water infusion and 6.5 ml/min for SDC and DNase steps. Following the end of the decellularization cycle, the livers were perfused with PBS with 1% antibiotic/antimycotic (Sigma, St Louis, MO) for 30 min and stored in the same buffer at 4 °C.

### Histology and immunohistochemistry

Samples were fixed for at least 24 h in 10% neutral buffered formalin solution (pH 7.4) at room temperature. Tissue was embedded in wax and sectioned at 4 μm. Sections were dewaxed in xylene and rehydrated using graded industrial denatured alcohol (IDA) before staining with Harris’s hematoxylin and eosin (H&E) (Leica Microsystems, Wetzlar, Germany), or Picro-Sirius Red (SR) (VWR International, Radnor, PA) or Miller’s Elastic with a SR counter stain (VWR International, Radnor, PA; Leica Microsystems, Wetzlar, Germany; Thermo Scientific, Waltham, MA).

For AA immunohistochemistry, 2 µm sections were de-waxed, rehydrated and endogenous peroxidase blocked with 0.6 % hydrogen peroxidase. Sections were then washed in PBS, and non-specific antibody binding was blocked by incubation in 2.5% v/v heat inactivated normal horse serum (Vector Labs, Burlingame, CA) for 30 min at room temperature. After washing, slides were incubated with anti-mouse SAA antibody Ab AF2948 (R&D Systems, Abingdon, UK) diluted in Dako Antibody Diluent (1/100) overnight at 4 °C. Slides were then incubated with IMMpress anti-goat polymer (Vector Labs, Burlingame, CA) for 30 min at room temperature, washed and incubated for 10 min with DAB/Metal (Thermo Scientific, Waltham, MA) diluted (1:10) in stable H_2_O_2_ substrate buffer. After terminating the reaction by washing, slides were counterstained with Myers hematoxylin for 30 s. Congo red staining was performed by the method of Puchtler et al. [15]. Cross-polarized light microscopy was used to reveal the pathognomonic green birefringence of Congo red stained amyloid.

### DNA quantification

Total DNA content within native tissue and acellular matrices was measured using the DNeasy Blood and tissue kit according to the manufacturer’s manual (Qiagen, Hilden, Germany). Briefly, specimens were digested with Proteinase K overnight, and DNA was purified on a spin column; purity and yield of purified DNA were assessed with spectrophotometric measurements at 260 and 280 nm.

### Scanning electron microscopy (SEM)

Samples were fixed in 2.5% v/v glutaraldehyde in 0.1 M phosphate buffer and left for 24 h at 4 °C. Following washing with 0.1 M phosphate buffer, samples were cut into segments of approximately 1 cm length and cryoprotected in 25% sucrose, 10% glycerol in 0.05 M PBS (pH 7.4) for 2 h, then fast frozen in nitrogen slush and fractured. Samples were then placed back into the cryoprotectant at room temperature and allowed to thaw. After washing in 0.1 M phosphate buffer (pH 7.4), the material was fixed in 1% OsO_4_/0.1 M phosphate buffer (pH 7.3) at 3 °C for 1.5 h and washed with 0.1 M phosphate buffer (pH 7.4). After rinsing with water, specimens were dehydrated in a graded ethanol-water series to 100% ethanol, critical-point dried using CO_2_ and finally placed on aluminum stubs using sticky carbon taps. The material was mounted to present fractured surfaces across the parenchyma to the beam and coated with a thin layer of Au/Pd (∼2 nm thick) using a Gatan ion beam coater. Images were recorded with a 7401 FEG scanning electron microscope (Jeol Ltd, Welwyn Garden City, UK).

### Protein composition of AA-*m*L_dec_ and AA-*m*L homogenates

Crude homogenates of livers from decellularized and untreated amyloidotic AA transgenic mice [12] (AA-*m*L_dec_ or AA-*m*L, respectively) were prepared according to the AEF extraction procedure from liver, as previously described [16]. Total protein content in homogenates was measured using Pierce BCA protein assay kit (Thermo Scientific, Waltham, MA) and 2 µg of total protein were analyzed using homogenous 15% PAGE under denaturing and reducing conditions for further proteomics and immuno-enzymatic analyses. For proteomic characterization, gel bands stained with colloidal Coomassie Blue were trypsin digested before analysis of the extracted peptide mixtures by MALDI-MS (AUTOFLEX III Smartbeam Bruker, Billerica, MA). Peptide calibration mixture (Bruker Daltonics, Billerica, MA) in the range between 1000 and 3500 Da was used as external standard.

Immunoblot analysis following 15% reducing SDS-PAGE was performed with primary goat polyclonal anti-mouse SAA1 antibody (0.2 µg/ml, R&D Systems, Abingdon, UK) and secondary polyclonal anti-goat IgG peroxidase conjugate (0.65 × 10-^3^ µg/ml, Dako, Glostrup, Denmark).

### Amyloid enhancing factor activity in AA-mL_dec_

Two groups of five wild-type female C57BL/6 mice (Biological Services Unit, UCL) aged 12-15 weeks and weighing 23–26 g received an injection of 0.2 ml of AA-*m*L_dec_ or AA-*m*L homogenates, followed by repeated subcutaneous injections of 10% w/v casein in 0.01 M carbonate buffer. Casein was given for 5 days per week from day 1 to 14. To evaluate amyloid load, a single intravenous tracer dose of ^125^I-labeled serum amyloid P component (SAP) [17] was administered on day 23. Whole body retention of ^125^I was assessed after 24 and 48 h, and histological grading of Congo red stained formalin-fixed, wax-embedded tissues was performed as previously described [18,19].

### Ethical approval

This study was ethically reviewed and approved by the UCL Royal Free Campus Ethics and Welfare Committee and the UK Home Office, and complied fully with European Directive 86/609/EEC.

## Results

### Perfusion–decellularization of normal and amyloidotic mouse livers

**Figure 1.**
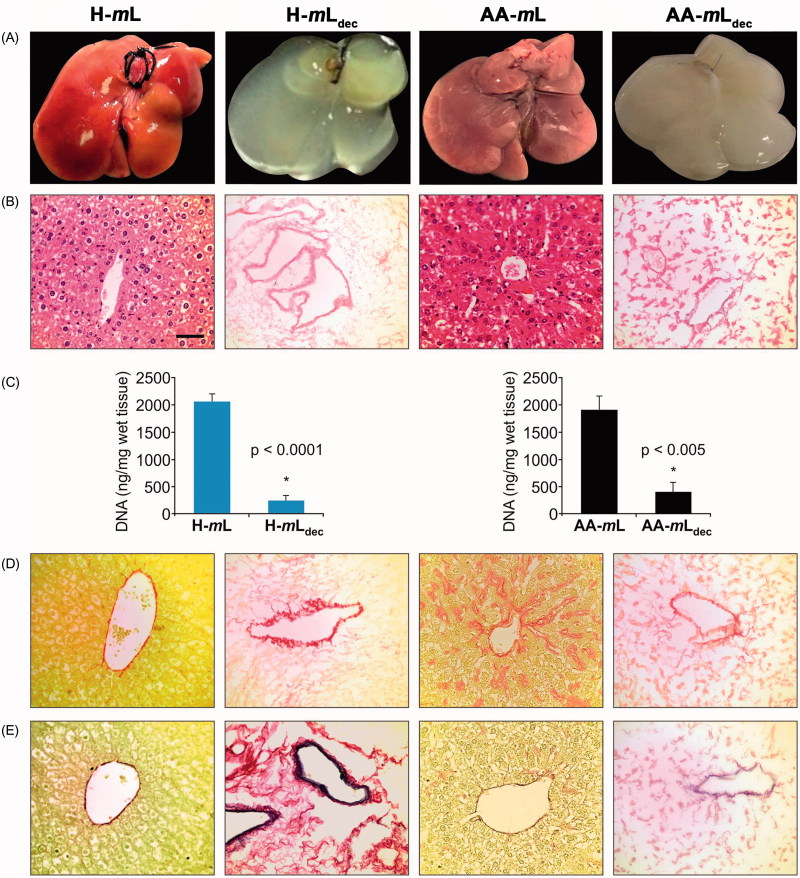
Characterization of liver tissue scaffolds. (A) Macroscopic appearance before and after the decellularization procedure of native healthy (H-*m*L), amyloidotic (AA-*m*L), decellularized healthy (H-*m*L_dec_) and amyloidotic (AA-*m*L_dec_). (B) Sections stained with H&E. (C) DNA quantification of mouse livers to show reduction of DNA after decellularization confirming removal of cells. (D) Sections stained with SR. (E) Elastin Van Gieson staining of mouse livers sections shows preservation of collagen and elastin after decellularization. Scale bar for 40× magnification: 50 µm.

The decellularization protocol, based on antegrade perfusion through the portal vein, was performed by sequential perfusion of water, detergent and DNAse. Decellularization of healthy and amyloidotic SAA transgenic mouse livers [12] was completed within 46 h perfusion. During and following decellularization, the whole liver gradually became increasingly translucent with the dissolution of cells (Figure 1A). Macroscopically, decellularized amyloidotic livers (AA-*m*L_dec_) retained an opaque appearance after decellularization (Figure 1A) when compared with the white translucent appearance of decellularized healthy livers (H-*m*L_dec_; Figure 1A). With the exception of minor regions in some cases, histological evaluation by H&E staining showed no evidence of cell bodies or cellular material, including nuclei in decellularized livers (Figure 1B). Spectrophotometric analysis demonstrated the removal of DNA from the tissue, confirming the efficiency of the perfusion decellularization method (Figure 1C). Nevertheless, the general liver tissue architecture appeared fully preserved as shown by SR staining for collagens (Figure 1D) and Van Gieson staining for elastin (Figure 1E). Importantly, the presence of amyloid in the organ did not affect the conduct or the efficacy of the perfusion and the resulting organ scaffolds from amyloidotic livers had the same hydrodynamic properties of pressure and flow rate as the scaffolds from normal livers.

### Amyloid is retained in the decellularized liver scaffold

**Figure 2.**
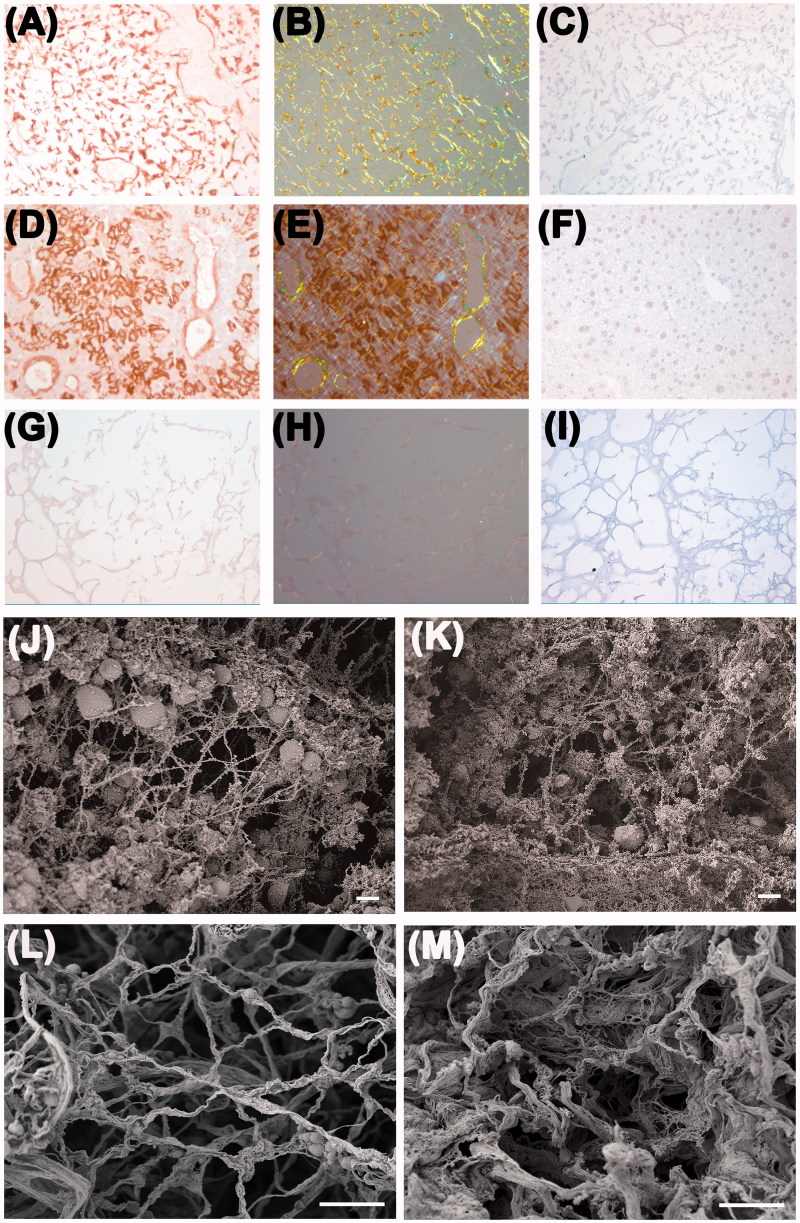
Amyloid in decellularized amyloidotic mouse liver. (A, B) Decellularized liver scaffold from a heavily amyloidotic mouse was stained by immunohistochemistry for SAA and with Congo red, and viewed under bright field illumination A and with crossed polarizers B respectively. (C) No primary antibody control for A. (D, E) Intact liver from an SAA expressing amyloidotic mouse stained for SAA and with Congo red and viewed under bright field, D, and crossed polarized light, E, showing amyloid in portal tracts. In addition, nascent SAA within the cytoplasm of hepatocytes gave strong immunohistochemical signal. (F) No primary antibody control for intact liver. (G, H) Decellularized healthy mouse liver did not stain with SAA antibody or Congo red viewed under bright field (G) or cross polarized light (H). (I) no primary antibody control for decellularized healthy mouse liver. (J, K) SEM images of native normal and amyloidotic livers respectively. (L, M) SEM images of decellularized healthy and amyloidotic liver, respectively. Scale bar 200 µm, A–I; 2 µm, J–K, and 10 µm, L–M.

Congo red staining for amyloid and immunohistochemical staining with anti-SAA antibodies of sections of decellularized amyloidotic liver were both strongly positive (Figure 2A–C) in comparison with sections of non-decellularized amyloidotic livers (Figure 2D–F) and decellularized healthy organs (Figure 2G–I). Amyloid was clearly retained in the decellularized scaffold and, in the example shown, the amyloid was present throughout the scaffold, associated with the stromal ECM, as well as with the portal tracts and central veins.

### Ultrastructural characterization of decellularized mouse liver

Scanning electron microscopy was used to evaluate the 3D-architecture and micro-structure of the ECM in both healthy and amyloidotic tissue before (Figure 2J and K) and after decellularization (Figure 2L and M). The overall extracellular liver tissue micro-structure was apparently perfectly maintained after decellularization. The borders of the regions corresponding to the hepatocyte–parenchyma were characterized by an organized fibrillar network in the decellularized healthy liver (Figure 2L). In contrast, the decellularized scaffold obtained from amyloidotic liver was markedly thickened and overlaid with amyloid which appears as granular spongy material occluding the fine porosity typical of normal ECM. Decellularization strikingly highlighted major differences between healthy and amyloidotic liver (Figure 2L and M) that were not evident in the untreated organs (Figure 2J and K).

### SAA fragments are the main components in AA-*m*L_dec_

**Figure 3.**
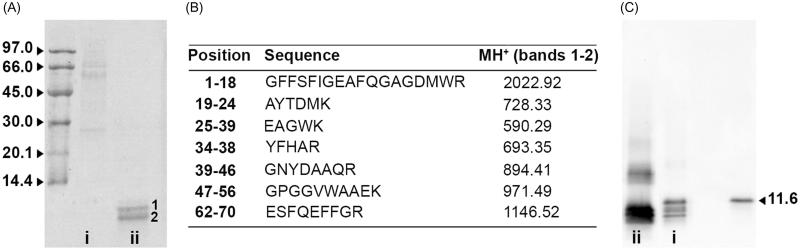
Characterization of AA mouse livers homogenates. (A) Total 2 µg of homogenates from AA-*m*L (lane i) and AA-*m*L_dec_ (lane ii) were separated in SDS 15% PAGE under reducing conditions. Apparent molecular weights for the marker are given in kDa. 1 and 2 indicate bands subjected to mass mapping analysis. (B) Tryptic peptides obtained by digestion of SDS-PAGE bands were analyzed by MALDI-MS. MH^+^ monoisotopic values (Da) are reported for each peptide. (C) Western blot analysis of AA-*m*L (i) and AA-*m*L_dec_ (ii) and standard serum mouse SAA_1_ (11.6 kDa) from the same strain of double transgenic mice [12] as AA-*m*L and AA-*m*L_dec_.

When equal amounts of protein from AA-*m*L_dec_ and AA-*m*L homogenates were run on a homogenous 15% SDS-PAGE and stained with colloidal Coomassie blue, a prominent doublet corresponding to AA amyloid protein [20] was observed only in the decellularized sample (Figure 3A). *In situ* trypsin digestion of the two main bands and MALDI-MS analysis of the extracted peptides confirmed that they both contained the 1–70 amino-terminal portion of murine SAA_1_ protein (Figure 3B). Western blot analysis confirmed the presence of SAA_1_ fragments in the AA-*m*L homogenate together with some full-length SAA protein (Figure 3C), the precursor of AA.

### Amyloid enhancing activity is preserved in AA-mL_dec_

**Figure 4.**
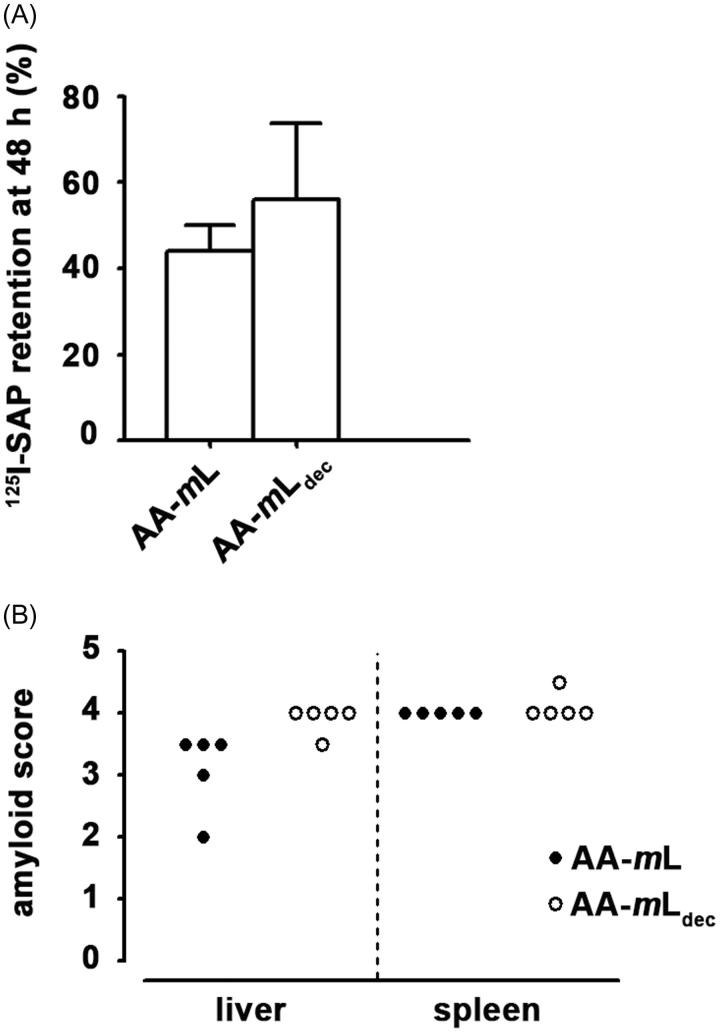
AA-*m*L_dec_ contains amyloid enhancing factor activity. (A) Amyloid, induced in groups of five C57BL/6 mice with homogenates of AA-*m*L_dec_ or AA-*m*L followed by repeated subcutaneous casein injections, was quantified by whole-body retention of ^125^I-SAP as percentage of the injected dose. (B) Histological grading of amyloid in both the spleen and the liver are shown.

It is known that tissue extracts from amyloidotic spleen/liver contain “amyloid enhancing factor” (AEF) activity [16]. A single injection of AA amyloid-containing tissue extracts accelerates amyloid deposition resulting from subsequent severe acute or prolonged inflammation, such as that evoked by repeated casein injections [16,21]. When groups of five mice received AA-*m*L or AA-*m*L_dec_, followed by casein injections, they had similar amyloid load as shown by ^125^I-labeled serum amyloid P component (^125^I-SAP) retention (Figure 4A) and histochemical Congo red staining of both spleen and liver (Figure 4B) [18,19].

## Discussion

We report for the first time the decellularization of a whole amyloidotic organ by intravascular perfusion with a defined detergent-enzymatic technique. Importantly, the decellularized liver preserves the amyloid deposits in the ECM and allows a clear analysis of the intimate relationship between ECM and amyloid. Indeed, cell removal highlighted the structure of the ECM and high resolution 3D microscopic analysis revealed that the constitutive tridimensional fibrillar network of ECM was disrupted and overlaid with amyloid leading to a marked modification of the very fine normal structure of the ECM.

The SEM images suggest that such extensive and intimate amyloid association with the ECM may have a profound effect on crucial biomechanical properties of the ECM such as organ elasticity and fluid permeability of the extracellular space. These observations are in partial agreement with previous studies in which SEM was used and results of decellularization of portions of amyloidotic tissue, mostly kidney, were reported [3,22]. These studies, while highlighting the intimate relationship between ECM and amyloid, report the presence of nodular aggregates with a granular surface rather than the more uniform distribution of lumps of amyloid along the tridimensional ECM structure shown in our study. It is likely that this difference in amyloid distribution is due to the different methods used to achieve tissue decellularization. Decellularization obtained by flow perfusion of the whole organ through the conserved natural vascular tree may preserve the innate relationship between amyloid and the 3D ECM meshwork. It is notable that amyloid in the scaffold revealed by perfusion decellularization retains the highly specific amyloid enhancing factor property of homogenates of the whole amyloidotic organs. This potent activity, attributable to amyloid fibrils themselves, or fragments thereof [23] has a crucial influence on the natural history of the disease once the first nuclei of amyloid are formed with existing fibrils having the capacity to catalytically accelerate the fibrillar conversion of protein precursors. AEF [16,23] prepared from decellularized amyloidotic liver maintains this catalytic property thus demonstrating that, with the conservation of amyloid fibril primary structure, tinctorial and microscopic features, decellularization did not affect the peculiar functional property of priming amyloid growth and propagation.

In conclusion, here we have demonstrated that a 3D ECM scaffold with a conserved vascular tree can be obtained from amyloidotic mouse liver with retention of the natural structure and specific amyloidogenic properties of the amyloid. Ongoing studies will provide a proteomic characterization of decellularized amyloidotic and non-amyloidotic liver. Once this study is completed, decellularized amyloid organs will represent an innovative experimental model to further investigate the disease and address crucial issues, such as, the relationship between the ECM and amyloid fibrils and the possible effects of natural fibrils on protein oligomerization as well on organ recellularization. High-resolution 3D images of amyloid in its natural environment may also be informative about the recently discovered putative expansive forces generated by fibril growth [24].

## Abbreviations

AA: amyloid A protein
(AA-*m*L): crude homogenates from non-decellularized AA amyloidotic mouse liver
AA-*m*L_dec_: crude homogenates from decellularized AA amyloidotic mouse liver
AEF: amyloid enhancing factor
ECM: extracellular matrix
H&E: hematoxylin and eosin
IDA: industrial denatured alcohol
IVC: inferior vena cava
PV: portal vein
SAA: serum amyloid A protein
SAP: serum amyloid P component
SDC: sodium deoxycholate
SEM: scanning electron microscopy
SR: picro-Sirius red.
